# Polynucleotides High Purification Technology (PN HPT^TM^) Injection Improves Pain Status and Functional Impairment in Hip and Shoulder Tendinitis

**DOI:** 10.3390/jcm14051404

**Published:** 2025-02-20

**Authors:** Paolo Gervaso, Massimiliano Minale, Niccola Funel

**Affiliations:** 1 Orthopedics Section, Division of Pavia, Bioanalysis Mutlidisciplinary Medical Center, Via Rivo Brignolo, Codevilla, 27050 Pavia, Italy; paologervaso@inwind.it; 2 Independent Researcher, Via R. De Blasio, Marigliano, 80034 Napoli, Italy; maxminale@hotmail.com; 3 Section of Laboratory Analysis, Division of Immunohematology, Department of Laboratory Diagnostics, Azienda Ospedaliera USL Nordovest, Via Lippi Francesconi, 55100 Lucca, Italy

**Keywords:** musculoskeletal disorders, tendinitis, novel therapeutic approach, polynucleotides, PN HPT^TM^, peritendinous injection, visual analog scale, functional impairment

## Abstract

**Background/Objectives:** Tendinopathy is the preferred term to describe various tendon pathologies, including paratendinitis, tendinitis, and tendinosis, in the absence of histopathological evidence in biopsy specimens. The management of tendinopathies is challenging; rest, physiotherapy (such as eccentric training), injections, shock waves, orthotics, medical therapy, and surgery are the main therapeutic options offered to the patient. The conservative treatment of tendinopathies is still difficult, but several options have been proposed, including the use of anti-inflammatory molecules. In this retrospective study, we aimed to assess the efficacy of a conservative approach in improving pain and functional improvement in hip bursitis (HB) and biceps tendinitis (BT) patients. **Methods:** A series of data concerning the application of Polynucleotides High Purification Technology (PN HPT^TM^) in 47 patients with BT and HB was analyzed. All patients received three bi-weekly injections of PN HPT^TM^ (T0–T2). Follow-up visits were performed at T3 (8 weeks from T2) and T4 (24 weeks from T2). Both the visual analog scale (VAS) for pain assessment and functional impairment (FI) scores were processed in the form of anonymized series for clinical improvement evaluations. **Results:** Statistically significant differences (*p* < 0.001) in pain reduction (−85%) and functional improvement (+86%) were found at the end of treatment. The levels of patient satisfaction (PS) and Clinical Global Improvement—Impression (CGI-I) were equal to 93% and 98%, respectively. According to the analyses, other patient data (e.g., gender, age, and BMI) did not appear to influence the positive treatment outcomes. **Conclusions:** The application of High Purification Technology (PN HPT^TM^) was shown to improve both pain and functional deterioration in patients with tendonitis in a similar manner to other conservative treatments. These retrospective analyses may open up new avenues for the implementation of conservative approaches in patients with tendinitis.

## 1. Introduction

### 1.1. Background

Tendinopathy is the preferred term to describe various tendon pathologies, including paratendinitis, tendonitis, and tendinosis, in the absence of histopathological evidence in biopsy specimens [1]. These conditions usually affect the shoulder and the hip. In particular, the prevalence of shoulder pain increases with age and physically demanding occupations, with a point prevalence of 26% and lifetime estimates of 67% [2], while hip problems, also known as trochanteric bursitis (TB), are commonly seen by sports medicine practitioners and affect as many as 5.6 patients per 1000 adults [3]. The management of tendinopathies is challenging, with rest, physiotherapy (such as eccentric training), infiltrations, shock waves, orthosis, medical therapy, and surgery being the main therapeutic options offered to patients [4,5]. In the acute treatment phase, the main objectives are the reduction in risk factors such as training errors, flexibility problems, and biomechanical abnormalities and the improvement in symptoms through rest and the application of ice, ultrasound, and laser therapy [6]. The conservative treatment of tendinopathies is still difficult. Generally, patients can expect their symptoms to improve within 3 to 12 months at most from the start of treatment.

### 1.2. Symptoms and Treatments

However, chronic symptoms persist in about a quarter of patients, even more than 10 years after treatment, and tendinopathies compromise both quality of life and physical activity [7]. Conservative treatments include platelet-rich plasma (PRP) [8] and corticosteroid infiltrations (CIs) [9]; a systematic review of clinical trials has shown that corticosteroids reduce pain in the short term but that this effect is canceled out in the medium–long term. After 3–6 months of ineffective conservative therapy, surgery should be considered [10]. In order to find safe and effective conservative treatments, the effects of pain on the musculoskeletal system, especially the part involving tendons and ligaments, such as rotator cuff tendinopathy, have recently been evaluated in several studies [10]. Indeed, both pain and functional impairment play fundamental roles in the relationship between patient and doctor.

### 1.3. Target of Clinical Management

Pain reduction and functional improvement are the goals in any stage of clinical management in patients affected by tendinitis [11]. However, conservative management does not seem to be an effective medium–long-term solution to achieve combined clinical improvement in the patient.

### 1.4. Clinical and Pathological Conditions

The two most common clinical conditions in regard to patient tendinitis are hip bursitis (HB) [12] and biceps tendinitis (BT) [13]. The former pathology includes lateral hip pain associated with greater trochanteric pain syndrome (GTPS) [14], which includes gluteus medius tendinopathy or tear, bursitis [15], and iliotibial band friction [16]. Posterior hip pain includes referred pain, such as low-back pain (LBP) [17], deep gluteal syndrome with sciatic nerve entrapment [18], ischiofemoral impingement [19], and hamstring tendinopathy [20]. The musculoskeletal pathologies affecting the upper limbs of the human body include diseases of the arm, such as BT [21]. Further, massive rotator cuff tears (MRCTs) represent a significant portion of all rotator cuff injuries, accounting for approximately 20–40% of all injuries [22,23]. In MRCT, the long head of the biceps tendon (LHBT) is frequently involved, resulting in anterior shoulder pain and loss of function associated with tendinitis, partial tear, or the subluxation of the shoulder [24,25]. In fact, these two pathologies (HB and BT) represent common clinical problems, including a variety of periarticular pathologies of both the hip [26] and the shoulder [27]. Consequently, the clinical management of these conditions often becomes mandatory [28,29]. There are two possible options for these two problems: the surgical approach [27,29,30,31] and the conservative one [31,32]. In order to treat these two musculoskeletal pathologies without performing surgical procedures, several conservative approaches are available in addition to the abovementioned PRP [8] and CIs [28], including non-steroidal anti-inflammatory drugs (NSAIDs) [12,33,34].

### 1.5. New Technical Approaches

Despite the many different therapeutic approaches based on injections with curative intent, the clinical features of both HB and BT are often unsolved in the long-term period. For this reason, physicians are looking for new approaches (surgical and non-surgical) in order to address these clinical problems [32]. A new approach may be represented by the use of injectable polynucleotides (PNs). These molecules are extracted from natural sources (fish intended for human consumption, such as trout gonads) by using a specific process developed by Mastelli Srl (Sanremo, Italy), referred to as High Purification Technology (HPT^TM^) [35]. The derivatives of enzyme degradation of polynucleotide chains (simple nucleotides, nucleosides, and nitrogen bases) are physiologically present in the extra-cellular environment and are useful substrates for cells [36]. Polynucleotides High Purification Technology (PN HPT^TM^) is already used in esthetic medicine, based on previous experience in other clinical applications [37]. Recent studies have demonstrated both the efficacy and safety of PN HPT^TM^ injections in both dermatology and esthetic medicine for skin rejuvenation [37,38]. Polynucleotides High Purification Technology (PN HPT^TM^) injections are also used against knee osteoarthritis (OA) [39]. Giarratana et al. [37] demonstrated the better efficacy of PN HPT^TM^ compared with hyaluronic acid (HA) injections. In fact, the same group of scientists reported significant positive effects for a long time, up to 26 weeks after treatment [39]. Intra-articular injection progressively enriches the synovial fluid of PNs and thus of nucleotides, purine, and pyrimidine bases, which cells can use to promote their vitality [36]. This healing process could also be reproduced in the context of tendinopathy, creating an ideal environment for the healing of damaged tendons. Currently, joint replacement surgery is considered the reference therapeutic approach for osteoarthritis in both the hip and knee [40].

### 1.6. Aim of Present Study

Given these premises, the aim of the present study was to assess the impact of PN HPT™ on pain reduction (VAS) and functional improvement (CGI-I) in patients with hip and shoulder tendinitis, compared to baseline values and other conservative treatments. Nonetheless, this analysis would like to ascertain whether the injections of Tropho Tend (7.5 mg/mL PN HPT^TM^; Mastelli, Sanremo, Italy) influenced the perception of both pain and functional impairment in subjects with HB and BT pathology.

## 2. Materials and Methods

### 2.1. General Aspects and Eligibility Criteria

Data were acquired over 4 months. The manipulation of data was performed by two authors (P.G. and N.F.) in order to build an “ad hoc” database including clinical and anonymized personal data. The informatic code assigned at the beginning of this phase of the study allowed us to maintain the anonymity of the patients. For data collection, all patients satisfied the following eligibility criteria: (1) they were affected by tendinitis, with particular preference for patients affected by hip bursitis (HB) and biceps tendinitis (BT); (2) they had not previously undergone reconstructive or conservative surgical treatments; (3) they had not benefited from infiltrative and/or conservative treatment; (4) they had no other concomitant pathologies within HB and BT; (5) they completed treatment with polynucleotides (PN HPT^TM^). A power calculation was performed using a study power of 90% and a 5% confidence interval. Based on this calculation, the minimum required sample size would be 45 participants.

### 2.2. Data Assessment

All data procedures were performed in agreement with the legal standards of human data manipulation as per the Helsinki Declaration of 1975, updated in 2000 and 2008. The patient data employed for clinical evaluation concerned a total of 5 visits (T0, T1, T2, T3, and T4). The final results were compared with respect to the baseline (T0). All patients included in the database first signed their informed consent; then, the treatments were performed, and complete clinical data were analyzed. The procedures regarding the informed consent presentation were as follows: (1) explanation of the treatment to the patient; (2) adhesion; (3) fill the document; (4) check the data; (5) signature; (6) association with anonymous code.

### 2.3. Treatment Documentation

**Figure 1:**
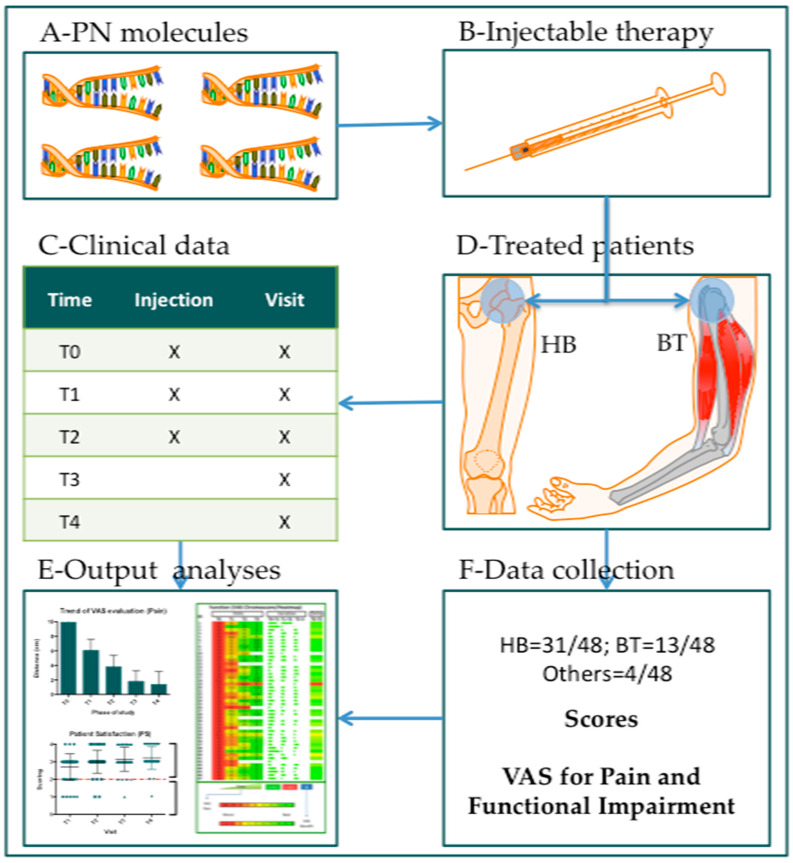
A working plan of the data analysis. All patients included in the data analysis study were treated with Polynucleotide HPT^TM^ (**A**) injections (**B**). The patients received three injections (**C**), and the clinical data were acquired five times (T0–T4). A total of 48 records were analyzed: 31 for hip bursitis (HB) and 13 for biceps tendinitis (BT) (**D**). The visual analog scale (VAS) score was used to quantify both pain and functional impairment variations (**F**). Significant statistical differences are reported in graph bars, graph plots, and heat maps (**E**).

All patients received Tropho Tend (7.5 mg/mL Polynucleotides High Purification Technology (PN HPT^TM^); Mastelli, Sanremo, Italy; Figure 1A) administered through the injection of the solution in the musculotendinous (MT) or osteotendinous (OT) junctions with a thin-gauge needle (typically 25–30 G; Figure 1B,C). Before the injection of the product, the target area was disinfected with alcohol or another antiseptic solution. The treatments consisted of 3 administrations of Tropho Tend: at T0—baseline visit; at T1—after 2 weeks; and at T2—after 4 weeks. All subjects were screened at the baseline visit (T0). Each subject was involved in the study for 7 months, for a total of 5 visits (T0 = baseline; T1 = after 2 weeks; T2 = after 4 weeks; T3 = after 8 weeks from T2; T4 = after 24 weeks from T2; Figure 1D). The results were compared with the baseline (T0; Figure 1E,F).

### 2.4. Score Evaluations

During each visit, all patients were evaluated for both pain and functional impairment by using a specific visual analog scale (VAS) from 0 to 10 cm, consisting of a line connecting the two extremes (0 = the absence of pain or of functional impairment; 10 = the worst possible pain or maximum functional impairment). The VAS has been previously used in order to evaluate the intensity of pain [41,42] and functional improvement, which is understood or interpretable as a reduction in the level of daily activities (ADL) [42,43]. The investigators evaluated the tendinopathy as improved or worsened compared with the baseline by using the Clinical Global Impression—Improvement (CGI-I) scale, a 7-point scale used to compare the patient’s condition between the visits and the baseline visit (T0). These analyses were based on the following parameters: visit data, patient feedback, patient satisfaction, and VAS analysis results. The CGI-I score was established according to a 7-point Likert scale, as the Likert scale is considered valid for assessing both the level and the value of certain characteristics of subjects [44]. The condition was judged as 1 = very much improved; 2 = much improved; 3 = minimally improved; 4 = no change; 5 = minimally worse; 6 = much worse; and 7 = very much worse. At the same time, patient satisfaction (PS) was also evaluated according to a 5-point scale. The PS values consisted of the following scores: 0 = very dissatisfied; 1 = dissatisfied; 2 = neutral; 3 = satisfied; 4 = very satisfied.

### 2.5. Statistical Procedures

#### 2.5.1. General Methodology

All data were listed and sorted by gender, analysis population, and, when appropriate, by visit number per subject. All the summary tables of the efficacy data were structured with a column for each target and were annotated with the total population size relevant to that table/treatment, including any missing observations, if any. All continuous variables were summarized using the following descriptive statistics: n (number), mean, standard deviation, median, maximum, and minimum. The frequency and percentages (based on the non-missing sample size) were reported for all categorical measures. The data generated in this study were recorded in a study-specific electronic system, and the original rows of data can be made available on demand. After the completion of data entry in the system and the resolution and closure of all discrepancies, the database was blocked to avoid any further modification. After quality checks, the SAS (Systems 9.4) format database was used for statistical analysis.

#### 2.5.2. Study Variables

For participants’ demographic and clinical data documentation, the following variables were documented: year of birth, age (years), gender (male/female), weight, height, BMI calculation, pain evaluation (VAS score), functional impairment evaluation (VAS score), questionnaire about patient satisfaction (PS), and questionnaire about patient Clinical Global Impression (CGI). In order to establish the level of patient satisfaction (PS), a 5-point Likert scale was used according to the experiences of other physicians [45].

#### 2.5.3. Analytical Test Application (ATA)

**Figure 2:**
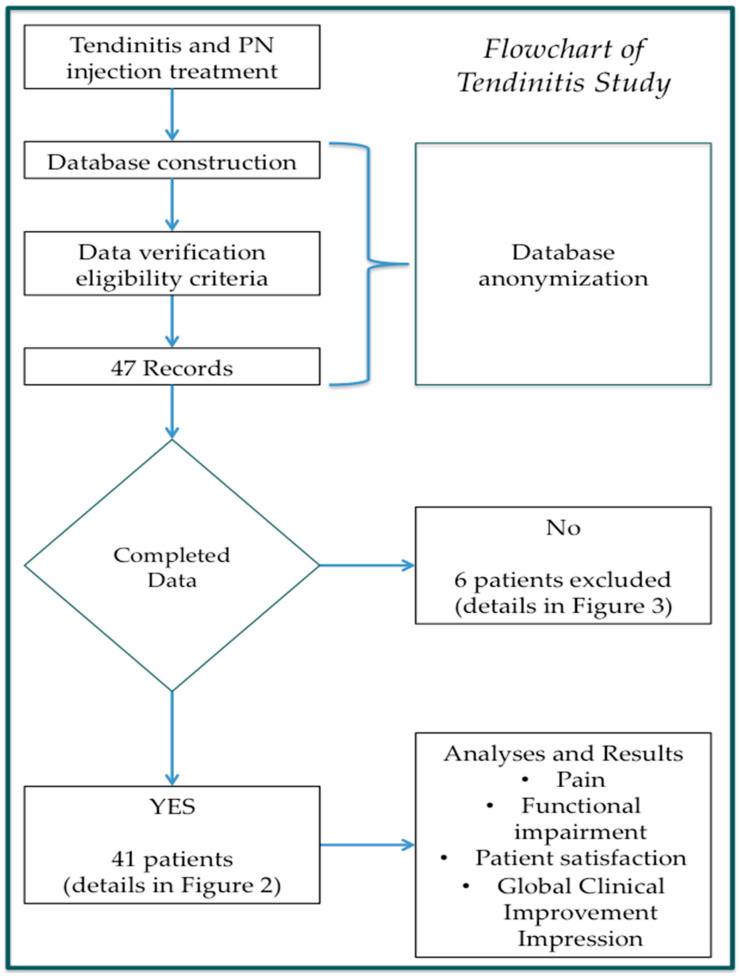
A flowchart of the study of tendinitis patients. The number for ‘Record’ represents the number of patients. A patient had bilateral HB.

The GraphPad 8.0 version for Apple Computer was used for statistical analysis (PRISM, San Diego, CA, USA). The Wilcoxon rank test was used to compare the differences between before and after the treatment in the degree of clinical improvements in tendinopathies and patient satisfaction. Non-parametrical tests (Kruskal–Wallis) were applied for group comparisons of the variables. One-way ANOVA was performed in order to compare the variation in the data across the visits. The final results were outlined with heatmap representations. All the parameters measured in this study were evaluated by using the classical descriptive statistics of the mean, SD, minimum and maximum (for quantitative variables), and frequencies (for qualitative variables). All statistical results were considered significant if the *p*-value was less than 0.05 (*p* < 0.05). The Shapiro–Wilk test was performed in order to determine whether the data were parametrically distributed. Both W- and *p*-values for the BMI data were calculated (W = 0.980 and *p* = 0.574). These values justified the implementation of parametric tests for the BMI analyses. The flowcahart of complete procedures is reported below (Figure 2).

## 3. Results

### 3.1. Demographic Description of Population

**Figure 3:**
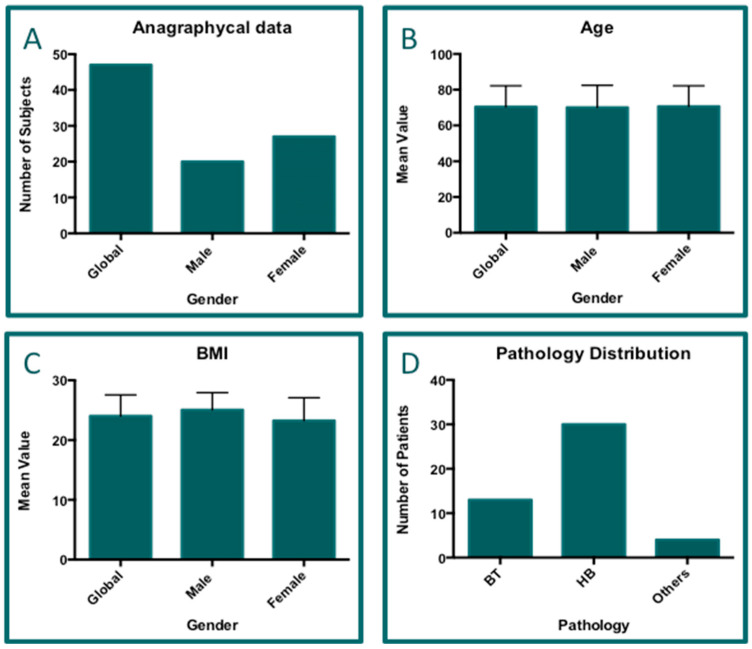
Multi-panel image concerning the demographic data of patients. Graph (**A**)—gender distribution; graph (**B**)—age distribution; graph (**C**)—BMI calculation; graph (**D**)—distribution of pathologies. Legend: BT = biceps tendinitis (13 cases); HB = hip bursitis (31 cases); others = miscellanea of other pathologies, comprising epicondylitis (1 case); epitrocletis (1 case); latissimus dorsi (1 case); plantar fasciitis (one case). One patient had two pathologies. The ANOVAs did not reveal significant differences among the groups reported in graphs (**B**,**C**) (*p* = ns).

The database, featuring a total of 47 different patients, contains the distributions of the demographic data (gender, age, BMI, and pathology), as reported in the multi-panel image below (Figure 3). Based on both weight and height acquisition data, the body mass index (BMI) was calculated for all the records included in the database. The patients were distributed as follows: males, 20 (42.55%), and females, 27 (57.45%) (Figure 3A). The mean age (Figure 3B) of the total cohort was 70.46 years (SD = 11.73), while the mean value of the BMI (Figure 3C) was equal to 24.0 points (SD = 3.52). Furthermore, the two most common pathologies were registered in this cohort of patients: biceps tendinitis (BT; 13/48; 27.08%) and hip bursitis (HB; 31/48; 64.58%; Figure 3D). One patient had two pathological sites (bi-lateral HB). The ANOVA and Student’s test revealed that there were no statistical differences in either age (Figure 3B) or BMI (Figure 3C) between males and females.

### 3.2. Description of Pathologies

**Figure 4:**
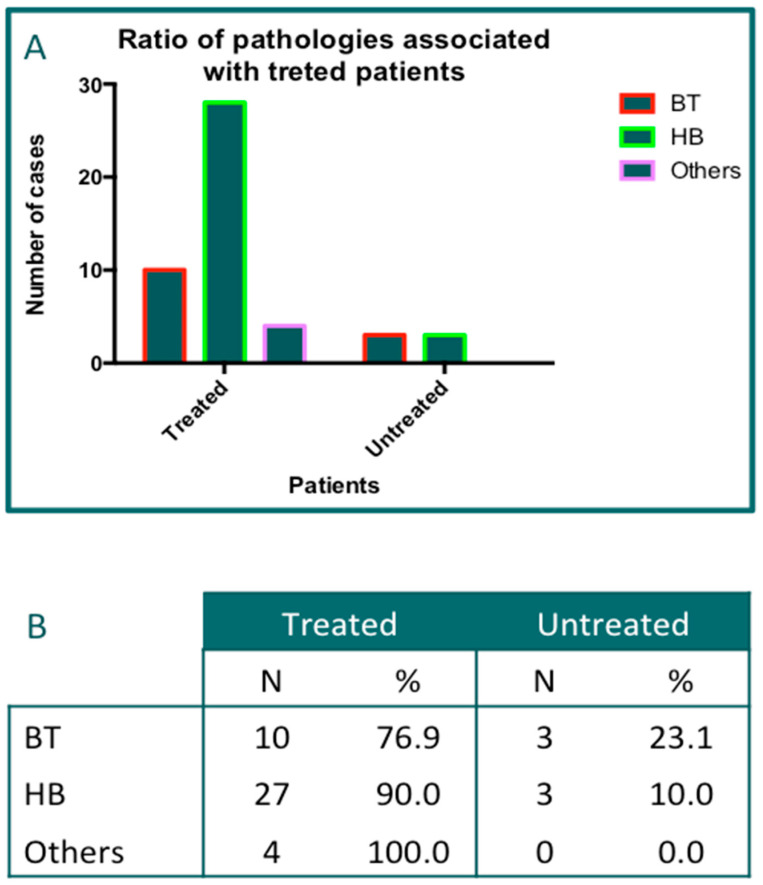
An analysis of the pathological conditions treated. (**A**): a bar graph showing the distribution of the pathologies. (**B**): a table reporting the percentages of completion of the treatment scheme. A total of 48 pathological sites were reported. One patient had bi-lateral HB.

A total of six types of different orthopedic pathologies were recorded at the beginning of data collection. However, in six cases, the follow-up was not performed due to the patients withdrawing from the study. In particular, in one case, the reasons were unknown; in four cases, there were unsuccessful results or dissatisfaction of the patient; in the last case, there were personal reasons. Thus, the frequencies of the pathologies associated with treated patients were evaluated according to the completion of the established protocol and are reported in Figure 4A. The most common pathological condition treated was hip bursitis (HB; 31/48; 64.58%), followed by biceps tendinitis (BT; 13/48; 27.08%). The data of the “miscellanea” group represented the remaining 8.42% of the pathological conditions (see Figure 3D). The data indicated that the treatment with PN HPT^TM^ injections was successfully completed in 41 of 47 patients. Thus, the figure for the proportion of completed treatments was 82.23% (Figure 4B).

### 3.3. Subjects with Incomplete Data

**Figure 5:**
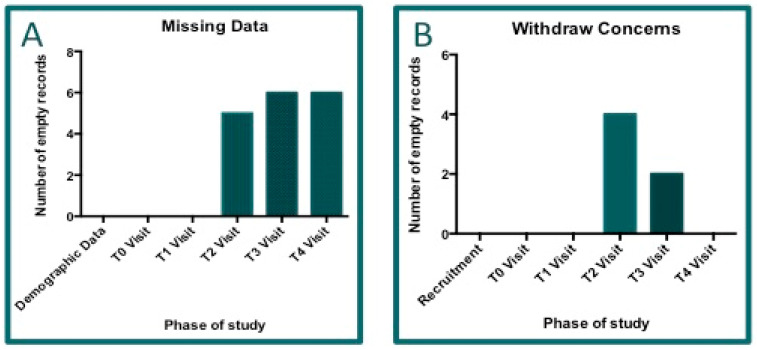
Missing data. Graph (**A**): A complete database was obtained by physicians for 41 out of 47 patients. Here, the missing data per visit are reported. Graph (**B**): The number of patients with incomplete data. Six of them were excluded from the clinical data analyses. There were five missing records for visit T2 and one for visit T3.

The information on absent data of clinical records is grouped in the two graphs reported below (Figure 5), where Figure 5A shows the total records distributed by visit, and Figure 5B indicates the number of patients not included in the global analyses due to the absence of data.

### 3.4. VAS Results (T0 Visit)

**Figure 6:**
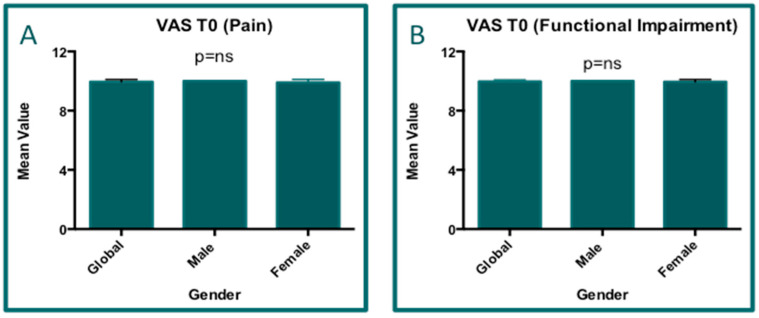
Graph (**A**): The VAS analysis results for pain (*p* > 0.05) reveal that there were no significant differences between the genders in the cohort of the study. Graph (**B**): the VAS analysis results for functional impairment (*p* > 0.05).

The baseline analyses for both pain and functional impairment (obtained based on the VAS scores) were performed with the Kruskal–Wallis (non-parametric) test. No statistical differences were found in both the pain and functional impairment rows according to the gender distribution. Both parameters showed mean values close to the maximum of the VAS score (mean VAS = 10). Below, the respective analysis results concerning pain (Figure 6A) and functional impairment (Figure 6B) at T0 are reported, according to which no statistical differences between males and females could be confirmed. Therefore, the results suggest that the differences in the patient-reported VAS scores might be dependent on the general condition of the patients and not influenced by gender, age, or BMI. Notably, data for HB and BT patients alone showed no difference in mean and standard deviation distribution (Table 1).

### 3.5. VAS Results (T0–T4 and Delta Analyses)

**Figure 7:**
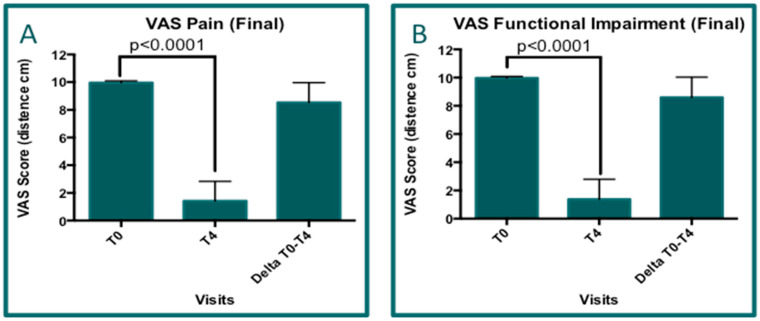
Graph (**A**): VAS analysis results for pain. Delta = 8.541 ± 1.429 (final visit T4 and delta value with respect to T0); *p* < 0.0001. Graph (**B**): VAS analysis results for functional impairment. Delta = 8.598 ± 1.441 (final visit T4 and delta value with respect to T0); *p* < 0.0001.

The data concerning the following visits (T0 and T4) for which it was possible to perform paired T-test analyses (Wilcoxon test) were available for 41 patients. The same analyses were performed for both pain and functional impairment VAS data (Figure 7). Regarding the difference observed in the pain score, the delta value was 8.537 ± 1.429 (Figure 7A), while the delta value for functional impairment was 8.598 ± 1.441 (Figure 7B). Furthermore, the pain and functional impairment reductions, in terms of VAS score difference (percentage), were equal to 85.68% and 86.19%, respectively. In particular, similar results were observed in looking for the two more representative groups for HB and BT (Table 2). These variations show significant statistical differences in both pain and functional impairment (*p* < 0.0001).

### 3.6. VAS Results (Global Trend)

**Figure 8:**
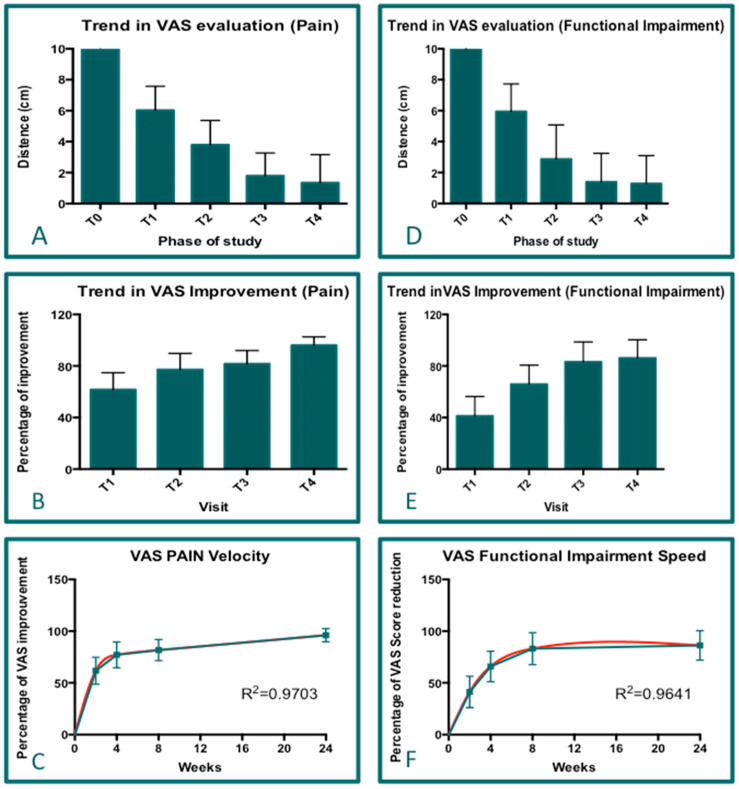
The cumulative analysis results of the VAS (pain and functional impairment) for all visits. Graphs (**A**–**C**) show the statistical data for pain, while graphs (**D**–**F**) show the statistical data for functional impairment. Graphs (**A**,**D**): The analysis results and description of the VAS (trends among the visits; *p* < 0.0001). Graph (**B**,**E**): The evaluation of VAS improvement (delta of the VAS with respect to T0; *p* < 0.0001). Graphs (**C**,**F**): The calculation of VAS improvement speed is based on the VAS score variation per week. The green line shows the connecting line among the points, while the red line demonstrate the R^2^ analysis (**C**,**F**).

The data concerning the visits (T0, T1, T2, T3, and T4) for which it was possible to perform ANOVAs (One-way) were available for 41 patients. All pairs of groups were compared against each other, and the same analyses were performed for both pain and functional impairment VAS data (Figure 8). The results reveal that the PN treatment showed great efficacy in improving both pain and functional impairment parameters, as according to the final results (T4), the mean improvement in the VAS score (for both parameters) was 85.89% (pain: 85.68%; functional impairment: 86.16%) with respect to T0 (Figure 8A,D; *p* < 0.0001). For both parameters (pain and functional impairment), there was a continuous and positive trend for the VAS score from visit T1 to visit T4 (Figure 8B,E; *p* < 0.0001). The delta values for VAS improvement (at visit T1) were 61.68% and 41.20% in pain and functional impairment, respectively. These results highlight the presence of two different speeds (Ss) of VAS improvement (VI; percentage) in the two parameters, pain and functional impairment (Figure 8C; R^2^ = 0.9703 and Figure 8F; R^2^ = 0.9641). By analyzing the speed of VAS improvement, it was possible to ascertain that the treatment accelerated the improvement in the functional impairment parameter faster (delta T4–T1: 44.96%) compared with the pain parameter (delta T4–T1: 35.42%; Figure 8B,D). In fact, it is possible to express the speed of VAS improvement (SVI) as the variation in the VAS per week (VI/w). Given that the time of visits covered a 22-week period (T1-T4), the SVI values for pain (1.56 VI/w) and functional impairment (2.04 VI/w) were calculated. Further, it is possible to express the acceleration as AVI (AVI/w^2^), and the following measurements were calculated for pain and functional impairment: 0.16 AVI/w^2^ and 0.20 AIV/w^2^, respectively. These values were obtained to express the percentage change in the VAS score per week; accordingly, a greater speed of improvement in the VAS (SVI) value was observed for the pain parameter, in which the improvement was equal to 4.00, while that observed in functional impairment was 3.59 (Figure 8C,F). Overall, by analyzing the results obtained for the pain parameter and for functional deterioration (measured with the VAS score), large statistical differences were observed and confirmed when comparing T0 vs. T4 (start point vs. end point). Therefore, the current results suggest that the VAS score is an adequate tool for evaluating improvements in pain status and functional impairments. Furthermore, it was observed that approximately 50% of large statistically significant differences in the improvement in the VAS score were observed in the early period of treatment (for example, at visits T1 and T2) (Table 3).

### 3.7. Questionnaire Results

**Figure 9:**
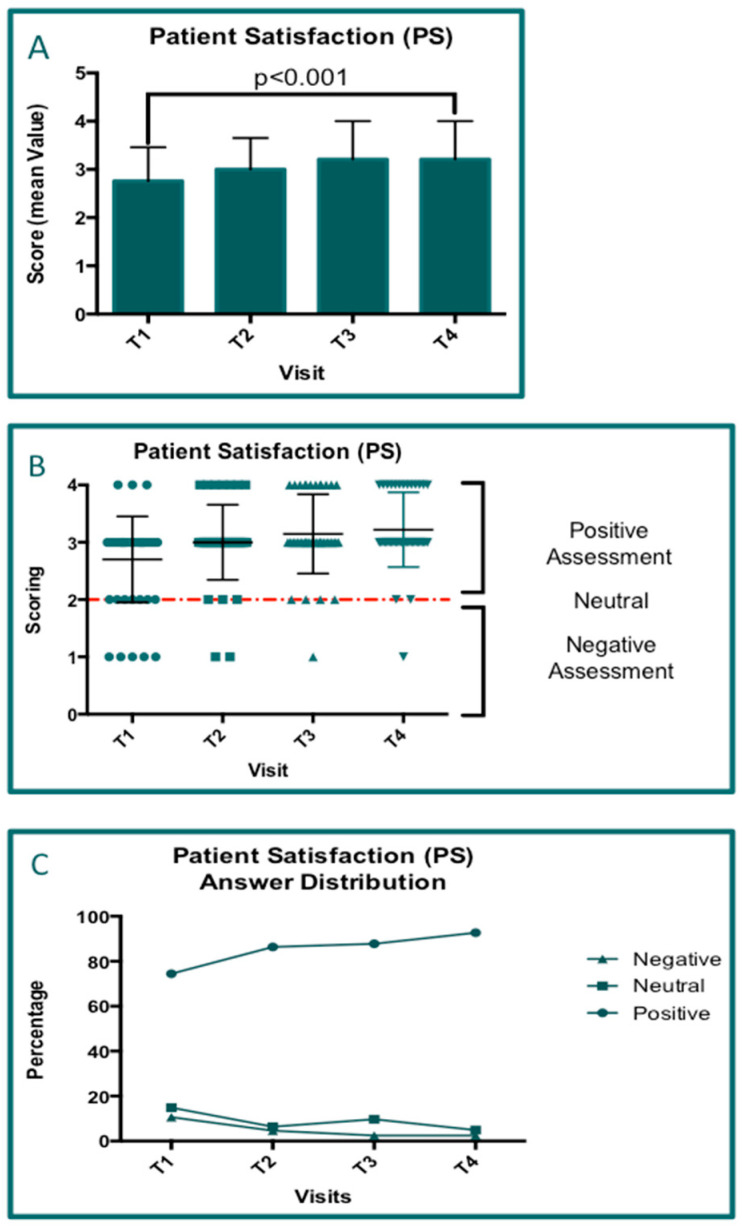
The results of the questionnaire concerning patient satisfaction (PS). (**A**) The bar graph shows the mean score value elaborated for each visit and the significative increase in PS over time. (**B**) A plot graph representing the distribution of the scores according to five levels of satisfaction grouping the patients who made positive and negative assessments. (**C**) A graph showing the percentages of the PS distribution across all visits.

**Figure 10:**
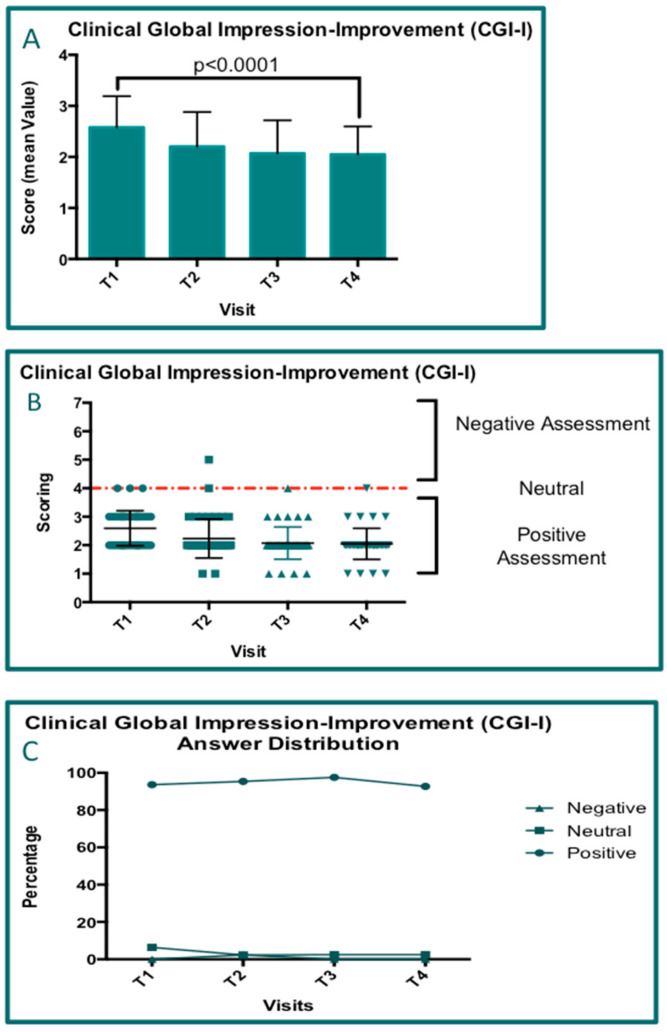
The results of the questionnaire concerning Clinical Global Impression—Improvement. (**A**) The bar graph shows the mean score value elaborated for each visit and the significative increase in CGI-I over time. (**B**) A plot graph representing the distribution of the scores according to seven levels of satisfaction grouping the patients who made positive and negative assessments. (**C**) A graph showing the percentages of the CGI-I distribution across all visits.

The data concerning the following visits (T1, T2, T3, and T4) for which it was possible to perform ANOVAs were obtained for 41 patients. The rows of data concerning the questionnaires for both patient satisfaction (PS; Figure 9) and Clinical Global Impression—Improvement (CGI-I; Figure 10) were analyzed by performing two-way ANOVA tests, and all groups were compared in pairs (Table 4). The same analyses were performed for both parameters (*p* < 0.0001). For both parameters (PS and GCI-I), progressive positive improvement was observed (Figure 9A and Figure 10A). In particular, the mean scores of the questionnaires visit by visit and at all checkpoints were calculated, and all values indicated a positive assessment by both patients and physicians. As reported in Figure 9B and Figure 10B, the distribution of the scores associated with positive assessments increases starting from T1 for both PS (Figure 9) and CGI-I (Figure 10). In particular, it is highlighted that the comprehensive positive values (at visit T4) for PS and CGI-I were 92.68% and 97.56%, respectively.

### 3.8. Heatmaps and Chromo-Score Representation

**Figure 11:**
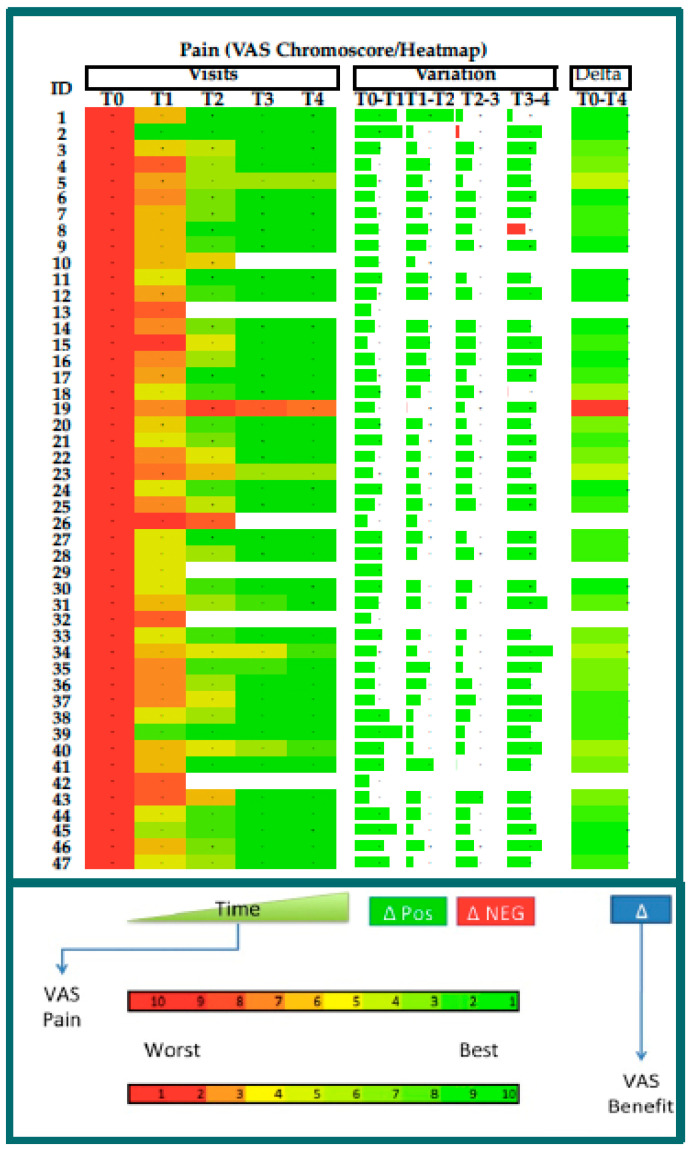
Heatmap representation of VAS data concerning pain analysis (*p* = 0.0001). Top left: singular interpretation of chromo-score for each visit. Top center: delta evaluation between consecutive visits, where width of bar means delta value. Green color represents positive changes (benefits). Red color represents negative differences (minus). Global variation in VAS (delta; top-right column) is represented according to chromo-score bar (bottom of panel).

**Figure 12:**
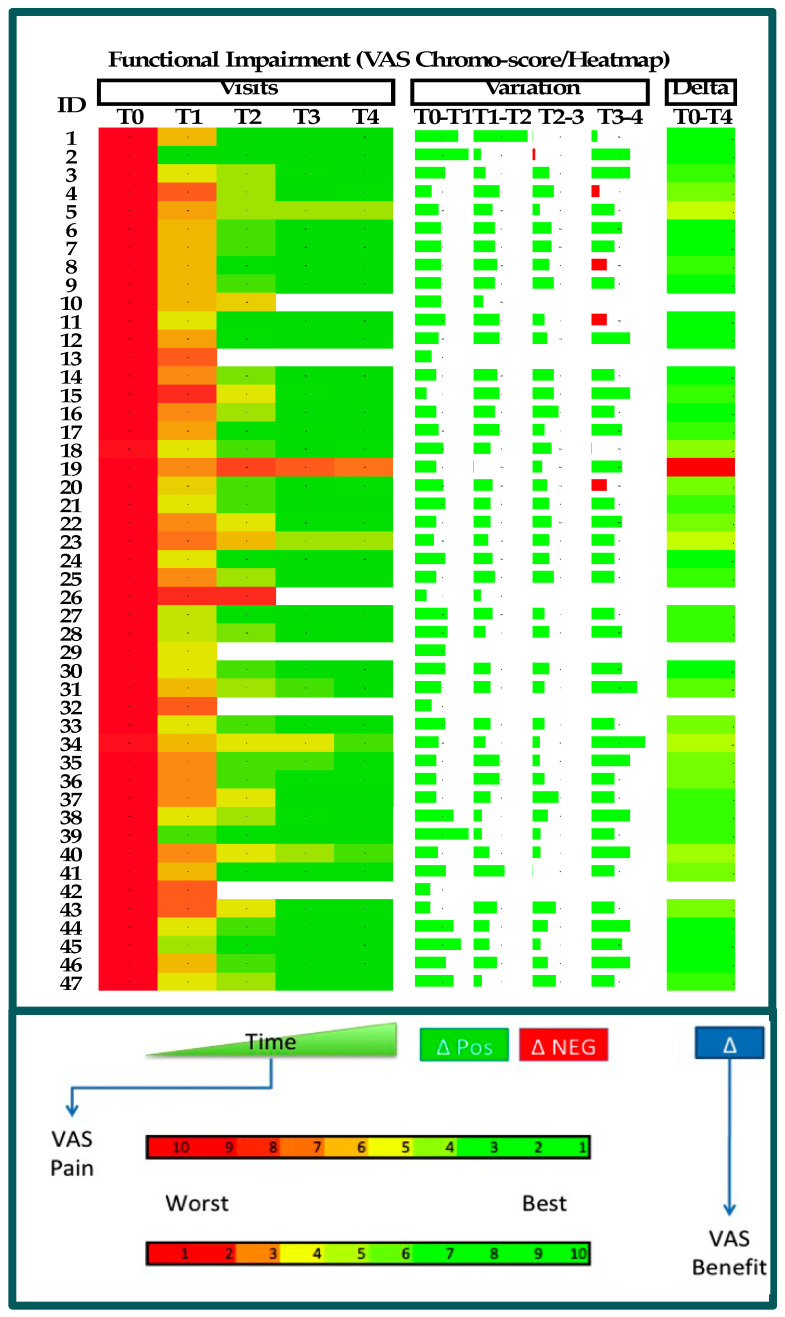
Heatmap representation of VAS data concerning functional impairment analysis (*p* = 0.0001). Top left: singular interpretation of chromo-score for each visit. Top center: delta evaluation between consecutive visits, where width of bar means delta value. Green color represents positive changes (benefits). Red color represents negative differences (minus). Global variation in VAS (delta; top-right column) is represented according to chromo-score bar (bottom of panel).

In order to explain the complex data of this study in a single picture, heatmap charts were used in order to highlight the progress of patients in terms of pain reduction and functional improvement in physical function. Both parameters for all the visits are reported in the figures below, where the chromo-scores range from red to green to represent the worst and best parameter values, respectively (Figure 11 and Figure 12). The heatmap shows that 40 out of 41 patients had a notable positive improvement after treatment, while 1 patient (#ID19) had a negative outcome in these analyses. However, it should also be mentioned that two patients (#ID02 and #ID41) experienced the greatest improvement in the VAS score for pain (Figure 11) and functional improvement (Figure 12) after just one injection. The green color used in Figure 11 and Figure 12 shows all the positive changes measured between one visit and the next. According to the pain analysis, in two cases (#ID02 and #ID08), the changes measured between T3 and T4 were negative, and in terms of functional improvement, five patients (#ID02, 4, 8, 11, and 20) showed negative results. In these cases, the changes in the VAS score between the two visits were very close to zero, and the computer algorithm assigned a negative value (red color). However, the final effect on the patients was not changed by this small difference, resulting in a global positive effect (green color).

### 3.9. Correlation Between BMI and Pathologies

**Figure 13:**
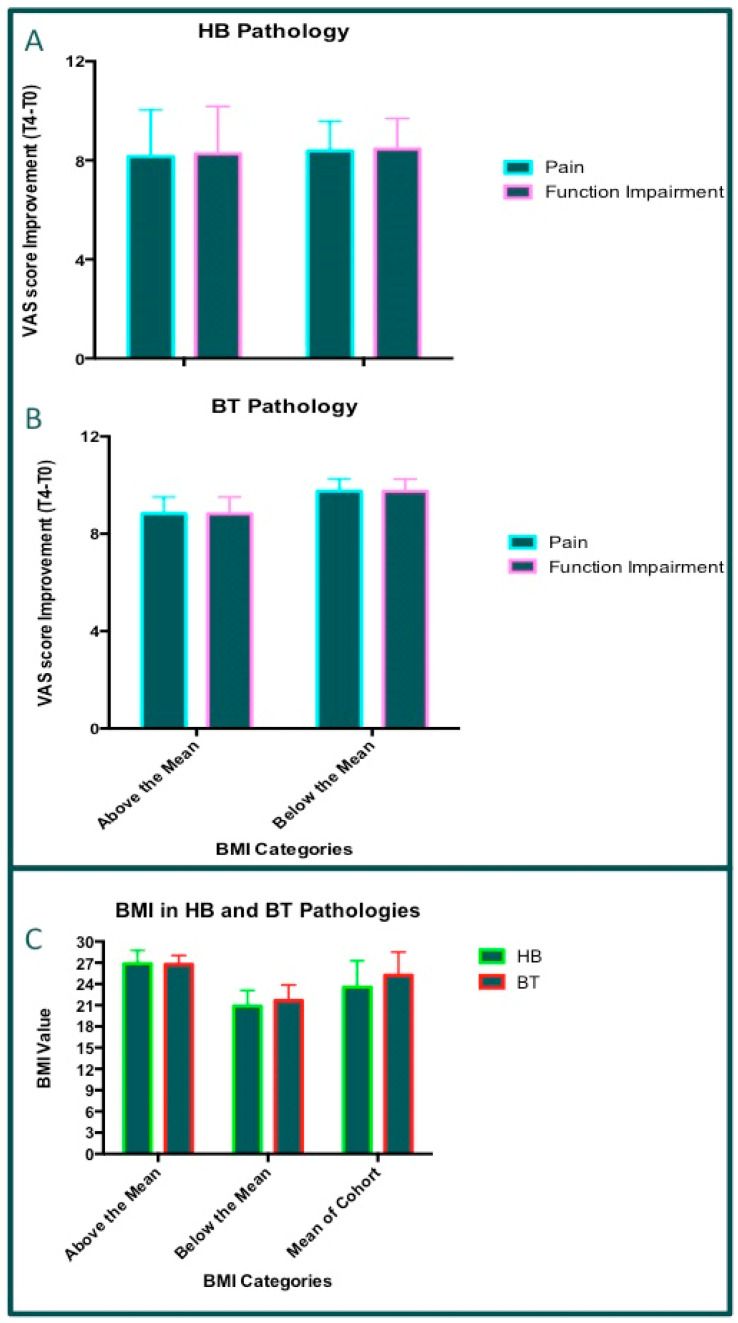
BMI and pathologies. Graph bars reporting patients sorted by BMI level (above or below mean): (**A**) hip bursitis (HB) pathology; (**B**) biceps tendinitis (BT) pathology; (**C**) HB and BT. ANOVA and multi-comparison T-test did not reveal significant values (*p* = ns).

According to both statistical reports, we tried to ascertain the presence of a difference in terms of body mass index (BMI) values (as reported in Figure 3C) in the patients treated for the considered pathologies. ANOVAs were carried out by comparing BMI and the two most frequent pathologies in patients treated with PN HPT^TM^, i.e., HB (hip bursitis) and BT (biceps tendinitis), as reported in Figure 3. The two pathologies were investigated separately, and the patients were sorted into two groups by the BMI value (upper or lower group, with respect to the mean value of BMI). In the BT group (10 patients), there were six patients showing a BMI value above the mean and four patients showing a BMI value below the mean. Furthermore, in the HB group, including 26 patients, there were 13 in both BMI groups (above and below the mean BMI value). The ANOVA tests did not reveal significant differences when comparing the mean BMI score values in both BT and HB patients (Figure 13A,B). Next, the global differences in the VAS score (T4–T0) were evaluated for both pain and functional impairment in the two most common pathologies treated with PN HPT^TM^ (BT and HB). According to the BMI partition, no significant statistical difference was discovered with ANOVA in these subgroups of patients (*p* = ns). The global VAS improvements in pain and functional impairment were the same in both groups of patients (sorted by BMI and pathology). This statistical evaluation is reported in Figure 13C. However, the statistical results indicate that BMI values lower than the mean were found in the group of patients showing greater improvement in both pain and functional impairment in BT (the differences were not significant). Finally, no significant differences were found in the HB subgroup of patients.

## 4. Discussion

### 4.1. The State of the Art

Tendinopathy is the commonly used term to describe various tendon pathologies that include paratendinitis, tendinitis, and tendinosis [1]. It has been reported that the Achilles tendon (AT) pathology alone constitutes 7–9% of total injuries in elite runners [46]. However, other tendinopathies should not be overlooked: a total of 1–2% of the general population has been reported to suffer from tendinopathy of the lateral elbow extensor tendon (LET) as a common origin [47], and 20% of all knee injuries have been diagnosed as patellar tendinopathies (PTs). Other common sites of tendinopathy include the proximal insertion of the hamstring, the rotator cuff tendons of the shoulder, and the trochanteric tendons [1]. Given the prevalence of these tendinopathies, the curative strategy in the treatment of tendon pathology remains an important point for clinical improvement in patients [1]. Conservative treatments represent the ideal choice for doctors to improve the clinical condition of patients without prolonging recovery times too much [7]. Such treatments include platelet-rich plasma (PRP) [8] and corticosteroid injections (CIs) [9]. However, a systematic review of clinical studies has shown that corticosteroids reduce pain in the short term but that this effect is canceled out in the medium–long term. After 3–6 months of ineffective conservative therapy, surgical intervention should be considered [10]. Another technical approach might be the injection of Polynucleotides High Purification Technology (PN HPT™).

### 4.2. The Rationale of This Study

The utility of polynucleotides (such as in PN HPT™) has been widely documented over the last decade in several studies conducted since 2014 [36,39,48,49,50,51]. Orthopedics is the medical field in which PNs have been most widely used. In particular, three different groups of physicians performed randomized clinical trials (RCTs) on patients with knee osteoarthritis (OA) [36,39,49] and rotator cuff syndrome (RCS) [50], comparing treatments with PN HPT™ versus those with hyaluronic acid (HA) [36,39,50]. A total of 242 patients were enrolled in these studies, and 121 of them were treated with PN HPT™ alone [36,39,49,50]. Notably, in two of the RCTs, the physicians demonstrated the non-inferiority of PN HPT™ treatments with respect to HA alone [39,49]. The comparison between the two treatments revealed a marked difference in terms of non-steroidal anti-inflammatory drug (NSAID) consumption, as a reduction in the latter was observed in the arm, including for PN patients 8 weeks earlier than in HA patients (at 10 weeks in PN HPT™ vs. 18 weeks in HA [39] and at 8 weeks in PN HPT™ vs. 16 weeks in HA [49]). The results demonstrated a positive impact of PN applications in order to reduce pain in the first six months of treatment both in patients with knee OA and in those with RCS [36,39,50]. Further, similar medical findings were reported by Guelfi et al. in 2020 [51], who prospectively studied 146 patients with knee OA and 56 patients with ankle OA. The authors concluded that the PN HPT™ treatment positively improved the patients’ WOMAC score after one month and that the positive effects remained stable for the following six months. Furthermore, they supported the hypothesis that PN HPT™ could behave similarly to high-molecular-weight HA, with the advantage of fewer injection difficulties [51]. In another study focused on hip OA [48], scientists retrospectively evaluated the application of PN HPT™ alone in 43 patients with hip OA. Pain analyses (based on the VAS approach) demonstrated a reduction in the score of −46.6% from the baseline at the six-month follow-up [48]. Further, the VAS score remained low after 24 (−51.4%) and 36 (−55.6%) months (with respect to the baseline assessment) [48]. No studies in which PN application, tendinitis, and VAS improvement were collectively considered were found. Based on the literature, both in terms of topics and areas of application of polynucleotides, we would like to suggest three points of novelty for our scientific proposal: (1) recent articles reporting only the results obtained with PN HPT^TM^ focus on osteoarthritis and skin rejuvenation [37,48,52,53]; (2) clinical applications of PN HPT^TM^ regarding tendinitis seem to be missing in the literature in orthopedic patients [48,51]; (3) the use of PN HPT^TM^ polynucleotide injection, in hip bursitis, seems to be a new element in the literature. In good faith, we did not find data concerning the following variables: PN HPT^TM^ [7.5 mg/mL], HB, and VAS evaluation.

### 4.3. Purposes and Evidence

Our series of retrospective data confirmed that the treatment with Polynucleotides HPT^TM^ injection improved pain and functional impairment in both HB and BT subjects (*p* < 0.0001). Further, the “final chromo-score data” confirm and support the results of the analyses for T0–T4. It is worth noting that the clinical data of both HB and BT patients might represent the pathological conditions of GTPS [14] and LHBT [24,25], as indicated in Figure 1D. In fact, the treatment based on PN HPT™ corresponded to the large trochanteric site [31] and the long head of the biceps [54]. The significant statistical values obtained for both pain and functional impairment in these musculoskeletal conditions indicate an important role of Polynucleotides HPT^TM^ (PN HPT™) in treating the pathologies investigated. In particular, the results obtained in the earlier phases of treatment (e.g., at T1 and T2) indicate that this treatment both has positive outcomes and shows early efficacy. Moreover, the trend in the VAS scores showed a constant reduction over time. The VAS score is an important parameter for evaluating both pain and functional improvements in osteo-articular (OA) and musculoskeletal pathologies [15,17,55]. In particular, it is important to note that (see the global chromo-score analyses) the mean of the delta score (T0 vs. T4) was important in both pain and functional impairment assessments. These statistically significant differences were sought for all tendinitis patients and in both groups analyzed separately (HB and BT). In the second-level analyses, it was observed that the acceleration and speed of VAS improvement were different for the two parameters investigated. Higher acceleration was observed for functional impairment, while higher speed was found for the pain parameter. Furthermore, only one subject (corresponding to 2.38% of the total patients included) did not show treatment benefits in terms of both pain and functional impairment parameters. The results of different studies in OA diseases seem to confirm a similar trend associated with the PN HPT™ treatment [36,39,48,49,55]. In addition, in a study focusing on joint hip replacement and hematological parameters, before and after surgery, no consolidation regarding functionality was reported [40]. Nevertheless, this patient was shown to derive constant benefits from treatment, but their final VAS score remained much higher with respect to the general treated population (# ID19; Figure 10 and Figure 11). On the other hand, two patients (#ID02 and #ID41) received the maximum benefits after only one injection at T1 and/or T2 (see heatmaps in Figure 10 and Figure 11). A total of 168 points of analyses (comparing all delta points for each patient) were performed for pain and functional impairment (see heatmap analyses). Only 1 (0.59%) and 4 (2.38%) delta points out of 168 were negative in the pain and functional impairment analyses, respectively. However, in these four patients, the final result (delta values for visits T0–T4) was also positive. Furthermore, at the end of the data analyses (which could be performed in 87.23% of 47 patients), a total of 40 subjects achieved fully positive VAS improvements in pain and functional impairment (Figure 10 and Figure 11). The most frequently used scales for assessing the improvement in tendon function in patients with hip and shoulder dysfunction are the Shoulder Pain and Disability Index (SPADI) [56] and Harris Hip Score (HHS) [57] scales, respectively. However, as this is a retrospective study, these data were not available. Nevertheless, we attempted to convert the VAS scores reported in the manuscript into values corresponding to these scales, based on previously reported conversion methods [58,59]. These converted data are provided in the Supplementary Materials. Tables S1 and S2 represent the SPADI and HHS extrapolation. Indeed, both SPADI and HHS were calculated for both BT and HB, respectively (Tables S3 and S4). The statistical analyses showed similar results, as did the VAS analyses highlighted previously (Figures S1 and S2). Notably, in order to analyze all six different pathologies present in the database, the use of VAS score appeared as the best choice. On the other hand, the questionnaires showed twin positive trends for patient satisfaction (PS) and Clinical Global Improvement—Impression (CGI-I) data. In these analyses, it was observed that more than 90% of responses fell in the category of positive assessment scores for both PS and CGI-I, while negative assessments were reported in less than 3% of the questionnaires’ answers. Nonetheless, the following summary of the variables investigated by VAS can be offered [56]: pain, functional impairment, patient satisfaction, and CGI-I indicated a positive trend after PN HPT^TM^ injection. Lastly, we found that variable personal data such as age, gender, and BMI had no influence on the treatment results mentioned above, nor did the pathologies considered (HB and BT).

## 5. Conclusions

The results obtained from the analysis of the present demonstrated the efficacy and safety of Tropho Tend (PN HPT^TM^) use in tendinitis patients, especially in both HB and BT pathologies. The PN HPT^TM^ treatment improved the pain and functional impairment levels of patients. The results of this retrospective clinical data analysis are in agreement with the other contributions in the literature. The further studies, including a control population (e.g., other type of treatment), could consolidate the results of the present study and increase the molecular know-how associated with PN HPT^TM^. In vivo experiments and subsequent analyses support the results illustrated in this report and the rationale of the studies mentioned above. Nonetheless, the results obtained from the cross-match between the BMI analyses and the patient pathologies reveal no impact of BMI on tendon healing. Overall, Polynucleotides HPT^TM^ might represent a new weapon against tendinitis.

## Figures and Tables

**Table 1:** Statistical data of Figure 6.

	Parameter	Groups				
		HB	BT	Global	Male	Female
Pain	Mean	9.954	10.000	9.968	9.975	9.963
	SD	0.160	0.00	0.124	0.102	0.133
	N	28	10	47	20	27
Functional Impairment	Mean	9.963	10.000	9.972	9.981	9.955
	SD	0.133	0.00	0.102	0.0.96	0.150
	N	28	10	47	20	27

Note: The data concerning both the HB and BT group were extracted from all groups of tendinitis patients.

**Table 2:** Statistical data of Figure 7.

	Parameter	HB Group		BT Group		All Groups		
		T0	Delta T4–T0	T0	Delta T4–T0	T0	T4	Delta T4–T0
Pain	Mean	9.954	8.315	10.000	9.200	9.968	1.427	8.541
	SD	0.160	1.553	0.000	0.753	0.124	1.408	1.429
	N	28	28	10	10	47	41	41
	**Parameter**	**HB Group**		**BT Group**		**Groups**		
		**T0**	**Delta T4–T0**	**T0**	**Delta T4–T0**	**T0**	**T4**	**Delta T4–T0**
Functional Impairment	Mean	9.963	8.107	10.000	9.200	9.976	1.378	8.598
	SD	0.133	1.208	0.000	0.753	0.109	1.418	1.441
	N	28	28	10	10	41	41	41

Note, the data concerning both the HB and BT group were extracted from all groups of tendinitis patients.

**Table 3:** Statistical data of Figure 8A,D.

	Parameter	Groups				
		T0	T1	T2	T3	T4
Pain	Mean	9.963	5.902	3.695	1.841	1.426
	SD	0.131	1.492	1.426	1.468	1.407
	N	41	41	41	41	41
Functional Impairment	Mean	9.975	5.865	3.475	1.682	1.378
	SD	0.109	1.516	1.478	1.544	1.417
	N	41	41	41	41	41

**Table 4:** Summary of statistical tests.

Analysis	Parameter	Values of Test (Kruskal–Wallis)				
		*p*-Value	H-Value	χ^2^-Value	η^2^-Value	Power
VAS Pain	T0	0.9987	0.002619	0.0026	−0.011	0.867
VAS FI	T0	0.9854	0.029390	0.0029	−0.011	0.867
VAS Pain	Delta T0–T4	<0.0001	24.6683	24.67	0.093	0.9171
VAS FI	Delta T0–T4	<0.0001	26.9532	26.95	0.100	0.9171
**Analysis**	**Parameter**	**Values of Test (One-Way ANOVA)**				
		***p*-Value**	**F-Value**	**df-Values**	**η^2^-Value**	**Power**
VAS Pain	Trend T0–T4	<0.0001	298.0	(4200)	0.720	0.8996
VAS FI	Trend T0–T4	<0.0001	291.5	(94,200)	0.740	0.8842

## Data Availability

All data obtained from this study are available for consultation. The data controller is Paolo Gervaso.

## Supplementary Materials

The following supporting information can be downloaded at https://www.mdpi.com/article/10.3390/jcm14051404/s1, Figure S1: Representation of SPADI results in BT patient. The SPADI was elaborated at T0 and T4 Visits. The statistical differences were evaluated by paired Student’s test. The mean SPADI score was equal to 100.00% at T0, while 8.00% at T4. The main delta value (T0–T4) was equal to 92%. The PN HPT^TM^ treatment reduced the SPADI in all BT patient; *p* < 0.0001; Figure S2: Representation of HHS results in HB patients. The HHS was elaborated at T1 and T4 Visits. The statistical differences were evaluated by paired Student’s test. The mean HHS was equal to 65.00% at T1, while 85.00% at T4. The main delta value (T0–T4) was equal to 20%. After PN HPT^TM^ treatments, 27 out of 28 (96.42.%) patients showed an improvement of HHS, while 24 out of 28 (85.71%) patients showed the good level of HHS after PN HPT^TM^ treatments; *p* < 0.0001.; Table S1: Calculation of SPADI; Table S2: Calculation of Hip HARRIS Score (HHS); Table S3: Results of SPADI in 10 BT patients; Table S4: Results of HHS in 28 HB patients.

## References

[1] Woodley B.L., Newsham-West R.J., Baxter G.D., Kjaer M., Koehle M.S. (2007). Chronic Tendinopathy: Effectiveness of Eccentric Exercise. Br. J. Sports Med..

[2] Hodgetts C.J., Leboeuf-Yde C., Beynon A., Walker B.F. (2021). Shoulder Pain Prevalence by Age and within Occupational Groups: A Systematic Review. Arch. Physiother..

[3] Brinks A., Van Rijn R.M., Bohnen A.M., Slee G.L., Verhaar J.A., Koes B.W., Bierma-Zeinstra S.M. (2007). Effect of Corticosteroid Injection for Trochanter Pain Syndrome: Design of a Randomised Clinical Trial in General Practice. BMC Musculoskelet. Disord..

[4] Millar N.L., Silbernagel K.G., Thorborg K., Kirwan P.D., Galatz L.M., Abrams G.D., Murrell G.A.C., McInnes I.B., Rodeo S.A. (2021). Tendinopathy. Nat. Rev. Dis. Primer.

[5] Van Der Vlist A.C., Winters M., Weir A., Ardern C.L., Welton N.J., Caldwell D.M., Verhaar J.A.N., De Vos R.-J. (2021). Which Treatment Is Most Effective for Patients with Achilles Tendinopathy? A Living Systematic Review with Network Meta-Analysis of 29 Randomised Controlled Trials. Br. J. Sports Med..

[6] Cardoso T.B., Pizzari T., Kinsella R., Hope D., Cook J.L. (2019). Current Trends in Tendinopathy Management. Best Pract. Res. Clin. Rheumatol..

[7] Childress M.A., Beutler A. (2013). Management of Chronic Tendon Injuries. Am. Fam. Physician.

[8] Mathew J., Sankar P., Varacallo M. (2024). Physiology, Blood Plasma. StatPearls.

[9] Mellor R., Bennell K., Grimaldi A., Nicolson P., Kasza J., Hodges P., Wajswelner H., Vicenzino B. (2018). Education plus Exercise versus Corticosteroid Injection Use versus a Wait and See Approach on Global Outcome and Pain from Gluteal Tendinopathy: Prospective, Single Blinded, Randomised Clinical Trial. Br. J. Sports Med..

[10] Dean B.J.F., Lostis E., Oakley T., Rombach I., Morrey M.E., Carr A.J. (2014). The Risks and Benefits of Glucocorticoid Treatment for Tendinopathy: A Systematic Review of the Effects of Local Glucocorticoid on Tendon. Semin. Arthritis Rheum..

[11] Stanos S.P., McLean J., Rader L. (2007). Physical Medicine Rehabilitation Approach to Pain. Anesthesiol. Clin..

[12] Seidman A.J., Taqi M., Varacallo M. (2024). Trochanteric Bursitis (Archived). StatPearls.

[13] Elser F., Braun S., Dewing C.B., Giphart J.E., Millett P.J. (2011). Anatomy, Function, Injuries, and Treatment of the Long Head of the Biceps Brachii Tendon. Arthrosc. J. Arthrosc. Relat. Surg..

[14] Redmond J.M., Chen A.W., Domb B.G. (2016). Greater Trochanteric Pain Syndrome. J. Am. Acad. Orthop. Surg..

[15] LaPorte C., Vasaris M., Gossett L., Boykin R., Menge T. (2019). Gluteus Medius Tears of the Hip: A Comprehensive Approach. Phys. Sportsmed..

[16] Reiman M.P., Goode A.P., Hegedus E.J., Cook C.E., Wright A.A. (2013). Diagnostic Accuracy of Clinical Tests of the Hip: A Systematic Review with Meta-Analysis. Br. J. Sports Med..

[17] Wilson J.J., Furukawa M. (2014). Evaluation of the Patient with Hip Pain. Am. Fam. Physician.

[18] Gómez-Hoyos J., Martin R.L., Martin H.D. (2018). Current Concepts Review: Evaluation and Management of Posterior Hip Pain. J. Am. Acad. Orthop. Surg..

[19] Hernando M.F., Cerezal L., Pérez-Carro L., Canga A., González R.P. (2016). Evaluation and Management of Ischiofemoral Impingement: A Pathophysiologic, Radiologic, and Therapeutic Approach to a Complex Diagnosis. Skeletal Radiol..

[20] Degen R.M. (2019). Proximal Hamstring Injuries: Management of Tendinopathy and Avulsion Injuries. Curr. Rev. Musculoskelet. Med..

[21] McDevitt A.W., Young J.L., Cleland J.A., Hiefield P., Snodgrass S.J. (2024). Physical Therapy Interventions Used to Treat Individuals with Biceps Tendinopathy: A Scoping Review. Braz. J. Phys. Ther..

[22] Burkhart S.S., Barth J.R.H., Richards D.P., Zlatkin M.B., Larsen M. (2007). Arthroscopic Repair of Massive Rotator Cuff Tears with Stage 3 and 4 Fatty Degeneration. Arthrosc. J. Arthrosc. Relat. Surg..

[23] Virk M.S., Cole B.J. (2016). Proximal Biceps Tendon and Rotator Cuff Tears. Clin. Sports Med..

[24] Abraham V.T., Tan B.H.M., Kumar V.P. (2016). Systematic Review of Biceps Tenodesis: Arthroscopic Versus Open. Arthrosc. J. Arthrosc. Relat. Surg..

[25] Friedman D.J., Dunn J.C., Higgins L.D., Warner J.J.P. (2008). Proximal Biceps Tendon: Injuries and Management. Sports Med. Arthrosc. Rev..

[26] Shemesh S.S., Moucha C.S., Keswani A., Maher N.A., Chen D., Bronson M.J. (2018). Trochanteric Bursitis Following Primary Total Hip Arthroplasty: Incidence, Predictors, and Treatment. J. Arthroplasty.

[27] Ardebol J., Ghayyad K., Pak T., Galasso L., Noble M., Kiliç A.Ī., Gonzalez-Morgado D., Menendez M.E., Denard P.J. (2024). Long Head of Biceps Tendon Management in the Setting of Massive Rotator Cuff Tears. JSES Rev. Rep. Tech..

[28] Ali M., Oderuth E., Atchia I., Malviya A. (2018). The Use of Platelet-Rich Plasma in the Treatment of Greater Trochanteric Pain Syndrome: A Systematic Literature Review. J. Hip Preserv. Surg..

[29] Ahn J., Kim J.-H., Shin S.-J. (2024). Arthroscopic Suprapectoral Biceps Tenodesis Provided Earlier Shoulder Function Restoration Compared with Open Subpectoral Biceps Tenodesis during the Recovery Phase. J. Shoulder Elbow Surg..

[30] Lustenberger D.P., Ng V.Y., Best T.M., Ellis T.J. (2011). Efficacy of Treatment of Trochanteric Bursitis: A Systematic Review. Clin. J. Sport Med..

[31] Giai Via R., Elzeiny A., Bufalo M., Massè A., Giachino M. (2024). Endoscopic Management of Greater Trochanteric Pain Syndrome (GTPS): A Comprehensive Systematic Review. Eur. J. Orthop. Surg. Traumatol..

[32] Alghamdi A.A., Althaqafi R.M.M., Babaier Y.H., Singer M.S., Assiri S., Aljohani B., Alghamdi F.A., Abdel Badie A. (2022). Clinical Outcomes of Long Head Biceps Tendinitis Treatment by a Semitenodesis Technique. Cureus.

[33] Le D.T., Shah S. (2024). Greater Trochanteric Bursa Injection. StatPearls.

[34] Shbeeb M.I., Matteson E.L. (1996). Trochanteric Bursitis (Greater Trochanter Pain Syndrome). Mayo Clin. Proc..

[35] Colangelo M.T. (2021). Polynucleotide Biogel Enhances Tissue Repair, Matrix Deposition and Organization. J. Biol. Regul. Homeost. Agents.

[36] Vanelli R., Costa P., Rossi S.M.P., Benazzo F. (2010). Efficacy of Intra-Articular Polynucleotides in the Treatment of Knee Osteoarthritis: A Randomized, Double-Blind Clinical Trial. Knee Surg. Sports Traumatol. Arthrosc..

[37] Cavallini M., Bartoletti E., Maioli L., Massirone A., Pia Palmieri I., Papagni M., Priori M., Trocchi G. (2021). As Members of the Polynucleotides HPT^TM^ Priming Board, Collegio Italiano delle Società Scientifiche di Medicina Estetica (Italian College of the Aesthetic Medicine Scientific Societies)—SIME, AGORÀ, SIES. Consensus Report on the Use of PN-HPT^TM^ (Polynucleotides Highly Purified Technology) in Aesthetic Medicine. J. Cosmet. Dermatol..

[38] Park K.Y., Seok J., Rho N.K., Kim B.J., Kim M.N. (2016). Long-Chain Polynucleotide Filler for Skin Rejuvenation: Efficacy and Complications in Five Patients: Efficacy & Complications of Polynucleotide Filler. Dermatol. Ther..

[39] Giarratana L.S., Marelli B.M., Crapanzano C., De Martinis S.E., Gala L., Ferraro M., Marelli N., Albisetti W. (2014). A Randomized Double-Blind Clinical Trial on the Treatment of Knee Osteoarthritis: The Efficacy of Polynucleotides Compared to Standard Hyaluronian Viscosupplementation. Knee.

[40] Sánchez-Romero E.A., Battaglino A., Campanella W., Turroni S., Bishop M.D., Villafañe J.H. (2021). Impact on Blood Tests of Lower Limb Joint Replacement for the Treatment of Osteoarthritis: Hip and Knee. Top. Geriatr. Rehabil..

[41] Čota S., Delimar V., Žagar I., Kovač Durmiš K., Kristić Cvitanović N., Žura N., Perić P., Laktašić Žerjavić N. (2023). Efficacy of Therapeutic Ultrasound in the Treatment of Chronic Calcific Shoulder Tendinitis: A Randomized Trial. Eur. J. Phys. Rehabil. Med..

[42] Özmen T., Koparal S.S., Karataş Ö., Eser F., Özkurt B., Gafuroğlu Ü. (2021). Comparison of the Clinical and Sonographic Effects of Ultrasound Therapy, Extracorporeal Shock Wave Therapy, and Kinesio Taping in Lateral Epicondylitis. Turk. J. Med. Sci..

[43] Ahmad Z., Siddiqui N., Malik S.S., Abdus-Samee M., Tytherleigh-Strong G., Rushton N. (2013). Lateral Epicondylitis: A Review of Pathology and Management. Bone Jt. J..

[44] Stevens S.S. (1946). On the Theory of Scales of Measurement. Science.

[45] Atkinson M.J., Sinha A., Hass S.L., Colman S.S., Kumar R.N., Brod M., Rowland C.R. (2004). Validation of a General Measure of Treatment Satisfaction, the Treatment Satisfaction Questionnaire for Medication (TSQM), Using a National Panel Study of Chronic Disease. Health Qual. Life Outcomes.

[46] Järvinen T.A.H., Kannus P., Paavola M., Järvinen T.L.N., Józsa L., Järvinen M. (2001). Achilles Tendon Injuries. Curr. Opin. Rheumatol..

[47] Gabel G.T. (1999). Acute and Chronic Tendinopathies at the Elbow. Curr. Opin. Rheumatol..

[48] Migliore A., Graziano R., Martin L.S.M., Sorbino A., Raichi M., Boni G. (2021). Three-Years Management of Hip Osteoarthritis with Intra-Articular Polynucleotides: A Real-Life Cohort Retrospective Study. J. Biol. Regul. Homeost. Agents.

[49] Zazgyva A., Gergely I., Russu O.M., Roman C., Pop T.S. (2013). Polynucleotides versus sodium hyaluronate in the local treatment of knee osteoarthritis. Acta Med. Transilv.

[50] Saggini R., Di Stefano A., Capogrosso F., Carniel R., Haidar Hassan K., Bellomo R.G. (2014). Viscosupplementation with Hyaluronic Acid or Polynucleotides: Results and Hypothesis for Condro-Synchronization. J. Clin. Trials.

[51] Guelfi M., Fabbrini R., Guelfi M.G. (2020). Intra-Articular Treatment of Knee and Ankle Osteoarthritis with Polynucleotides: Prospective Case Record Cohort vs Historical Controls. J. Biol. Regul. Homeost. Agents.

[52] Araco A., Araco F., Raichi M. (2023). Clinical Efficacy and Safety of Polynucleotides Highly Purified Technology (PN-HPT®) and Cross-linked Hyaluronic Acid for Moderate to Severe Nasolabial Folds: A Prospective, Randomized, Exploratory Study. J. Cosmet. Dermatol..

[53] Lim T.S., Liew S., Tee X.J., Chong I., Lo F.J., Ho M.J., Ong K., Cavallini M. (2024). Polynucleotides HPT for Asian Skin Regeneration and Rejuvenation. Clin. Cosmet. Investig. Dermatol..

[54] Richards J.A., Haralson W.G., Woodard D.R., Nuelle C.W., DeFroda S.F. (2024). Arthroscopic Suprapectoral Biceps Tenodesis: A Knotless, Onlay, All-Suture Anchor Technique. Arthrosc. Tech..

[55] Cenzato N., Crispino R., Russillo A., Del Fabbro M., Tartaglia G.M. (2024). Clinical Effectiveness of Polynucleotide TMJ Injection Compared with Physiotherapy: A 3-Month Randomised Clinical Trial. Br. J. Oral Maxillofac. Surg..

[56] Breckenridge J.D., McAuley J.H. (2011). Shoulder Pain and Disability Index (SPADI). J. Physiother..

[57] Nilsdotter A., Bremander A. (2011). Measures of Hip Function and Symptoms: Harris Hip Score (HHS), Hip Disability and Osteoarthritis Outcome Score (HOOS), Oxford Hip Score (OHS), Lequesne Index of Severity for Osteoarthritis of the Hip (LISOH), and American Academy of Orthopedic Surgeons (AAOS) Hip and Knee Questionnaire. Arthritis Care Res..

[58] Shousha T., Alowais F., Arumugam A. (2022). Cross-Cultural Adaptation and Validation of the Arabic Version of the Simple Shoulder Test in the United Arab Emirates. PLoS ONE.

[59] Chandrasekaran S., Gui C., Walsh J.P., Lodhia P., Suarez-Ahedo C., Domb B.G. (2017). Correlation Between Changes in Visual Analog Scale and Patient-Reported Outcome Scores and Patient Satisfaction After Hip Arthroscopic Surgery. Orthop. J. Sports Med..

