# Supplementary material for: Polynucleotides High Purification Technology (PN HPTTM) Injection Improves Pain Status and Functional Impairment in Hip and Shoulder Tendinitis
Source: J Clin Med. 2025 Feb 20;14(5):1404. doi: 10.3390/jcm14051404 (PMC11901081; doi:10.3390/jcm14051404)
Supplement: Supplementary file 1 [file jcm-14-01404-s001.zip › jcm-3459038-supplementary.pdf]

Materials and Methods

Both the Shoulder Pain and Disability Index (SPADI) and the Harris Hip Score (HHS) are designed for clinical assessment of the shoulder and hip, respectively. Looking at the previously analyzed data set, possible values of SPADI and HHS were extrapolated for patients with BT and HB, treated with PN HPT. The SPADI evaluates the average score between pain and disability in percentage, based on the values of the VAS scale provided by the patient, in a questionnaire of thirteen answers. The maximum achievable score (severe clinical condition) is evaluated with 100%. Reductions of this percentage indicate an improvement in the patient's clinical conditions. The HHS questionnaire is also composed of two parts: The first one concerning pain (maximum score obtainable (44 points) and a second part concerning functionality (maximum score 47 points). The final score of this evaluation system is given by the sum of the maximum score is 91 points. The final result is expressed as a percentage. A higher score indicates a better clinical condition of the patient. To calculate the SPADI we used the data regarding both pain and disability, reported by VAS values in percentages, in agreement with the formulas used for the SPADI [58,59]. To calculate the HHS, instead, we used the results of the VAS for the evaluation of pain, while for disability we used the values reported through the Likert-scale for the evaluation of the CG-I. In this case the points associated with both pain and disability, where associated following the range established by HHS questionnaire. The conversion methods of these parameters are reported in the two tables below: SPADI (Table S1 and HHS Table S2).

Table S1: Calculation of SPADI

Conversion of VAS to SPADI Score looking for the Pain		Conversion of VAS to SPADI Score looking for the functionality	
VAS Values	SPADI %	VAS Values	SPADI %
1	10%	1	10%
2	20%	2	20%
3	30%	3	30%
4	40%	4	40%
5	50%	5	50%
6	60%	6	60%
7	70%	7	70%
8	80%	8	80%
9	90%	9	90%
10	100%	10	100%

The final SPADI score was obtained as mean value reported (percentage) among the pain and disability.

Table S2: Calculation of Hip HARRIS Score (HHS)

Conversion of VAS to Harris looking for the Pain		Conversion of Likert Scale to Harris Score looking for the functionality	
VAS Values	Harris points	CGI-I likert Scale	Harris Points
1	44	1	47
2-3	40	2	46
4-5	30	3	37
6-7	20	4	28
8-9	10	5	18
10	0	6	9
		7	0

The final HHS score was obtained a sum of Harris point concerning the pain and Harris point regarding the disability

Results.

Table S3: Results of SPADI in 10 BT patients.

	T0			T4			T0-T4		
	PAIN	Disability	Mean	PAIN	Disability	Mean	PAIN	Disability	Mean
1	100,00%	100,00%	100,00%	5,00%	5,00%	5,00%	95%	95%	95,00%
2	100,00%	100,00%	100,00%	0,00%	0,00%	0,00%	100%	100%	100,00%
3	100,00%	100,00%	100,00%	0,00%	0,00%	0,00%	100%	100%	100,00%
4	100,00%	100,00%	100,00%	5,00%	5,00%	5,00%	95%	95%	95,00%
5	100,00%	100,00%	100,00%	10,00%	10,00%	10,00%	90%	90%	90,00%
6	100,00%	100,00%	100,00%	0,00%	0,00%	0,00%	100%	100%	100,00%
7	100,00%	100,00%	100,00%	10,00%	10,00%	10,00%	90%	90%	90,00%
8	100,00%	100,00%	100,00%	20,00%	20,00%	20,00%	80%	80%	80,00%
9	100,00%	100,00%	100,00%	20,00%	20,00%	20,00%	80%	80%	80,00%
10	100,00%	100,00%	100,00%	10,00%	10,00%	10,00%	90%	90%	90,00%
Mean	100,00%	100,00%	100,00%	8,00%	8,00%	8,00%	92,00%	92,00%	92,00%

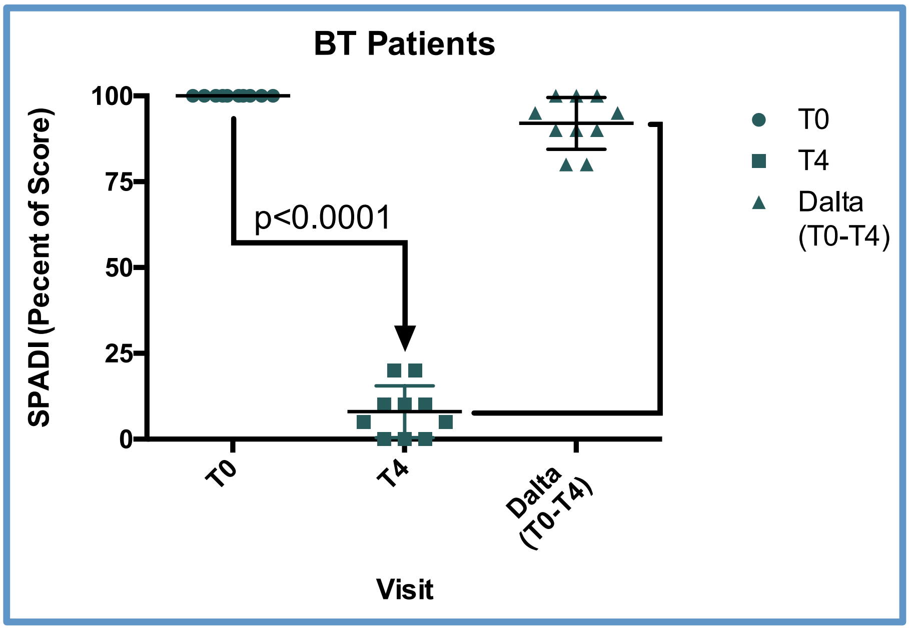

Figure S1: Representation of SPADI results in BT patient. The SPADI was elaborated at T0 and T4 Visits. The statistical differences were evaluated by paired Student's test. The mean SPADI score was equal to 100.00% at T0, while 8.00% at T4. The main delta value (T0-T4) was equal to 92%. The PN HPT™ treatment reduced the SPADI in all BT patient; p<0.0001.

Table S4: Results of HHS in 28 HB patients.

	T1				T4			
	Pain	Disability	SUM	Result	Pain	Disability	SUM	Result
1	30	46	76%	Fair	40	46	86%	Good
2	10	37	47%	Poor	40	46	86%	Good
3	20	37	57%	Poor	44	46	90%	Excellent
4	20	46	66%	Poor	44	46	90%	Excellent
5	20	37	57%	Poor	44	46	90%	Excellent
6	30	46	76%	Fair	44	46	90%	Excellent
7	20	37	57%	Poor	44	46	90%	Excellent
8	30	46	76%	Fair	40	46	86%	Good
9	20	37	57%	Poor	10	28	38%	Poor
10	30	37	67%	Poor	40	46	86%	Good
11	20	37	57%	Poor	30	37	67%	Poor
12	20	37	57%	Poor	44	46	90%	Excellent
13	30	46	76%	Fair	44	46	90%	Excellent
14	30	46	76%	Fair	44	46	90%	Excellent
15	30	46	76%	Fair	44	46	90%	Excellent
16	20	46	66%	Poor	40	46	86%	Good
17	30	37	67%	Poor	40	37	77%	Fair
18	20	37	57%	Poor	40	46	86%	Good
19	30	46	76%	Poor	40	37	77%	Fair
20	20	37	57%	Poor	40	46	86%	Good
21	20	37	57%	Poor	44	46	90%	Excellent
22	30	46	76%	Poor	44	46	90%	Excellent
23	40	28	68%	Poor	44	46	90%	Excellent
24	20	37	57%	Poor	40	37	77%	Fair
25	10	37	47%	Poor	40	46	86%	Good
26	30	46	76%	Fair	44	47	91%	Excellent
27	30	46	76%	Fair	44	47	91%	Excellent
28	20	46	66%	Poor	44	47	91%	Excellent

The HHS interpretation was assed though the following intervals of score, according the HHS procedure: Poor HHS <70%; Fair 71%<HHS<79%; Good 71%<HHS<79%; Excellent HHS>90.

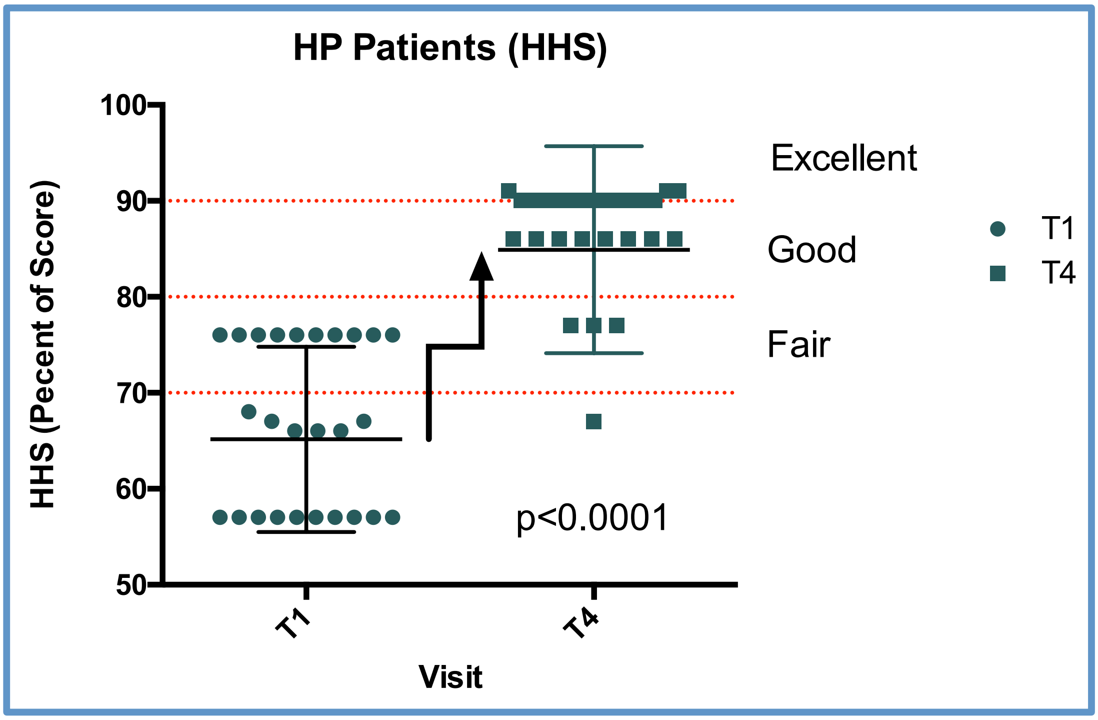

Figure S2: Representation of HHS results in HB patients. The HHS was elaborated at T1 and T4 Visits. The statistical differences were evaluated by paired Student's test. The mean HHS was equal to 65.00% at T1, while 85.00% at T4. The main delta value (T0-T4) was equal to 20%. After PN HPT™ treatments, 27 out of 28 (96.42.%) patients showed an improvement of HHS, while 24 out of 28 (85.71%) patients showed the good level of HHS after PN HPT™ treatments; p<0.0001.
